# A Close Examination of the Use of Systolic Time Intervals in the Calculation of Impedance Derived Cardiac Autonomic Balance and Regulation

**DOI:** 10.3389/fnins.2021.625276

**Published:** 2021-04-30

**Authors:** Cameron R. Wiley, Vida Pourmand, Julian F. Thayer, DeWayne P. Williams

**Affiliations:** ^1^Department of Psychological Science, University of California, Irvine, Irvine, CA, United States; ^2^Department of Psychology, Western Washington University, Bellingham, WA, United States

**Keywords:** cardiac autonomic balance, cardiac autonomic regulation, heart rate variability, pre-ejection period, left ventricular ejection time

## Abstract

Traditionally, impedance derived measures of cardiac autonomic balance (CAB) and regulation (CAR) are calculated using indices of heart rate variability (HRV) that primarily reflect parasympathetic nervous system activity (e.g., high-frequency HRV | HF-HRV) and pre-ejection period (PEP; a systolic time interval and measure of sympathetic activity). However, HF-HRV and PEP are considered measures of chronotropic and inotropic cardiac influence, respectively. Left ventricular ejection time (LVET) is a systolic time interval that reflects sympathetic chronotropic influence, and therefore may be a more appropriate measure for calculating CAB and CAR compared to PEP. Thus, the current study evaluates both PEP and LVET in the calculation of CAB and CAR. Data from 158 healthy participants (mean age = 19.09 years old, SD = 1.84 years) were available for analyses. CAB and CAR values were calculated using both HF-HRV and the root mean square of successive differences, in addition to both PEP and LVET, in accordance with previously established guidelines. Analyses showed that correlations were significantly weaker between CAB and CAR calculated using LVET for both HF (*z* = 5.12, *p* < 0.001) and RMSSD (*z* = 5.26, *p* < 0.001) than with PEP. These data suggest that LVET, compared to PEP, provides better “autonomic space” as evidenced by a lack of correlation between CAB and CAR computed using LVET. We stress that future research consider calculating CAB and CAR using chronotropic measures for both parasympathetic and sympathetic activity, as doing so may yield more accurate and independent measures of cardiac autonomic activity compared to a mixture of inotropic (i.e., PEP) and chronotropic (i.e., HF-HRV) measures.

## Introduction

The dynamic between the parasympathetic and sympathetic branches of the autonomic nervous system is a multifaceted one that is implicated in psychological and physiological processes and health (Sleight, 1997; Thayer et al., 2009). Good health is generally marked by a relative equilibrium between the parasympathetic and sympathetic branches, referred to as autonomic balance (Thayer and Friedman, 1997; Malliani, 2005). Conversely, poor health is linked to autonomic imbalance, which is characterized by hyperactive sympathetic activity and hypoactive parasympathetic activity (Malliani et al., 1994; Thayer et al., 2010b). Therefore, examining the association between cardiac autonomic activity, health outcomes, and psychological factors is of interest to many psychologists and physicians alike. In this effort, impedance derived measures of cardiac autonomic balance (CAB) and regulation (CAR) have been developed (Berntson et al., 2008). Traditionally, both CAB and CAR are calculated using respiratory sinus arrythmia or high frequency heart rate variability (HF-HRV; an index of heart rate variability and measure of parasympathetic activity) and pre-ejection period (PEP; a systolic time interval and measure of sympathetic activity) (Berntson et al., 2008; Singh et al., 2009; Kreibig et al., 2012; Bylsma et al., 2015). However, HF-HRV is considered a measure of chronotropic influence, defined as control of the heart via the sinoatrial node (Thayer et al., 2010a). In contrast, PEP is considered a measure of inotropic influence, defined as myocardial contractility (Levy, 1997). Thus, it is important to consider the calculation of CAB and CAR using indices of chronotropic influence for both parasympathetic and sympathetic measures. The left ventricular ejection time (LVET) is a systolic time interval that reflects sympathetic chronotropic influence, and therefore may be a superior measure (compared to PEP) for calculating CAB and CAR (Stemmler, 1993; Uijtdehaage and Thayer, 2000; Thayer and Uijtdehaage, 2001). Thus, the current study investigates both PEP and LVET in the calculation of CAB and CAR, and we highlight implications for how these differential calculations may impact psychophysiological research.

### Autonomic Balance and Health

Autonomic nervous system imbalance, or an increase in sympathetic activity coupled with a decrease in parasympathetic activity, has been associated with poorer physiological health outcomes including metabolic abnormalities (Licht et al., 2013) and cardiovascular disease risk factors (i.e., hypertension, diabetes) (Thayer et al., 2010b), as well as worse psychological outcomes, including anxiety (Friedman and Thayer, 1998), depression (Stone et al., 2020), and increased levels of daily stress (Mitchell et al., 2017). Due to its importance in health research, there have been several attempts to accurately measure cardiac autonomic balance and regulation over the years using various physiological measures. Berntson et al. (2008) proposed two indices of cardiac autonomic activity using impedance cardiography known as Cardiac Autonomic Balance (CAB) and Cardiac Autonomic Regulation (CAR). CAB is defined as the reciprocal balance between parasympathetic and sympathetic nervous system activity, while CAR is defined as the total activity of both branches. CAB and CAR can be calculated using indices of parasympathetically mediated HRV (e.g., the root mean square of successive differences [RMSSD], HF-HRV) and impedance derived systolic time intervals (i.e., pre-ejection period [PEP]) as an index of sympathetic activity (Berntson et al., 2008; Williams et al., 2017). Both CAB and CAR have been used as indices of autonomic balance and activity in a myriad of studies, showing associations with affective responses (Kreibig et al., 2012), psychopathologies (Bylsma et al., 2015; Stone et al., 2020), stress (Gump et al., 2011; Mitchell et al., 2017), inflammatory markers (Singh et al., 2009; Alen et al., 2020), and physiological health (Berntson et al., 2008; Vrijkotte et al., 2015). For example, a history of myocardial infarctions and type 2 diabetes diagnoses are more likely to be linked to low levels of CAR and CAB, respectively (Berntson et al., 2008), while lower CAB has also been shown to be associated with increased levels of inflammatory cytokines such as interleukin-6 and tumor necrosis factor alpha (Alen et al., 2020).

### Chronotropic vs. Inotropic Cardiac Influence

Autonomic influences on the heart can differ based on whether activation occurs at the sinoatrial (SA) node or the atrioventricular (AV) node. Autonomic nervous system activation at the SA node results in control of heart rate, known as chronotropy, which is associated with several cardiac measures including RMSSD (Thayer et al., 2010a). Among these measures is the left ventricular ejection time (LVET), a systolic time interval reflective of sympathetic activity (Stemmler, 1993; Thayer and Uijtdehaage, 2001). LVET is defined as the duration of the left ventricle to eject blood corresponding to the opening and closing of the aortic valve. More specifically, LVET refers to the interval between the B- and X-point on the dZ/dt waveform (Sherwood et al., 1990; Lozano et al., 2007). On the other hand, autonomic stimulation at the AV node results in changes in myocardial contractility, known as inotropy (Levy, 1997). A common inotropic measure is PEP, also a systolic time interval reflective of sympathetic activity, defined as the duration between initial ventricular depolarization and opening of the aortic valve. More specifically, PEP reflects the interval from the onset of the ECG Q-wave to the onset of left ventricular ejection (the interval preceding LVET) (Sherwood et al., 1990; Berntson et al., 2004).

Whereas both LVET and PEP are systolic time intervals that reflect sympathetic activity, the physiological foundations of these measures differ significantly. Therefore, a closer examination of the calculation of both CAB and CAR using PEP and LVET is warranted. Berntson et al. (1991) even acknowledged this potential issue in an earlier article, stating: “Moreover, in view of the highly specific patterns of autonomic activity that can be seen across organ systems, measures of the two autonomic divisions should be derived from the same organ. Finally, even chronotropic and inotropic influences on the heart, for example, are mediated by separate efferent pathways that may be subject to differential central control. Consequently, indices should be optimally derived from the same functional dimension of the target organ” (pp. 482-483).

### The Autonomic Space Model

**FIGURE 1:**
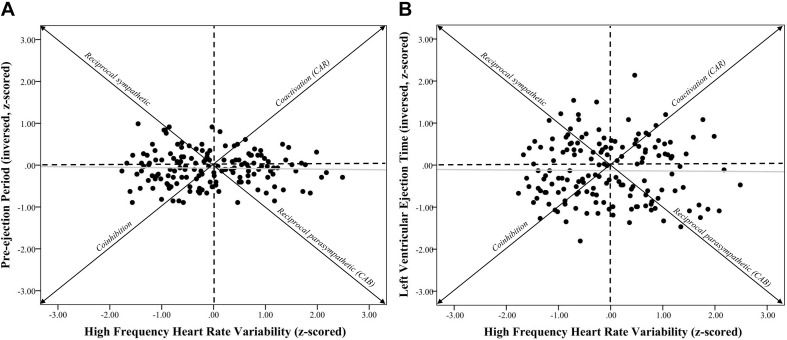
Systolic time intervals and high frequency heart rate variability scatterplots. *Note*. **(A)** shows a scatterplot between pre-ejection periods (PEP *z*-scored and inversed, see section “METHODS” for details) and high frequency heart rate variability (HF-HRV z-scored) (*r* = –0.03, *p* = 0.73). **(B)** shows a scatterplot between left ventricular ejection time (LVET inversed and z-scored) and HF-HRV (*r* = –0.01, *p* = 0.90). Individuals in the coinhibition quadrant would show lower CAR scores, while individuals in the coactivation quadrant would show higher CAR scores. Individuals in the reciprocal sympathetic quadrant would show lower CAB scores, while individuals in the reciprocal parasympathetic quadrant would show higher CAB scores.

Further exploration of the differential autonomic contributions of various cardiovascular measures led to the development of the Autonomic Space Model (Berntson et al., 1993), which proposed that chronotropic control of the heart via parasympathetic and sympathetic influence can vary reciprocally, independently, or coactively; laying the foundation for the future development of CAB and CAR. The autonomic “space” in question refers to the transformations that take place between psychophysiological antecedents and autonomic outflows (e.g., reciprocal, independent, or coactive), and between autonomic outflows and functional effects on target organs (i.e., chronotropic and inotropic influences on the heart) (Berntson et al., 1994b). The varying modes of autonomic control that the Autonomic Space Model describes can be illustrated using a bivariate model where the x-axis represents independent sympathetic control using a normalized sympathetic measure of cardiac activity (i.e., *z*-scores of PEP values) and the *y*-axis represents independent parasympathetic control using a normalized parasympathetic measure of cardiac activity (i.e., *z*-scores of HF-HRV). The graphical space within these axes can be divided into four quadrants that represent the modes of autonomic activity (reciprocal sympathetic, reciprocal parasympathetic, coactivation, and co-inhibition; see Figure 1 for example). Overall, this model provided a more comprehensive conceptualization of the flexibility of the autonomic nervous system and also serves as an additional way to examine the influence of different parasympathetic = and sympathetic measures on CAB and CAR. Importantly, one piece of such conceptualization, however, is that CAB and CAR are not significantly related. In other words, these various autonomic states as defined by CAB and CAR values can be *independent* from one another. For example, individuals could conceivably be higher in CAB, but not necessarily CAR. This is important, as CAB and CAR are thought to differentially predict cardiac disease states (e.g., myocardial infraction, diabetes; Berntson et al., 2008) and thus, CAB and CAR values should not be related or dependent on one another.

### Present Study

Given the importance of the autonomic nervous system in linking psychological and physiological health, it is crucial that the dynamic between its two branches be conceptualized in a way that optimally and accurately captures pure parasympathetic and sympathetic nervous system activity. Taking this into account, the purpose of this paper is to evaluate both PEP and LVET in the calculation of CAB and CAR. Specifically, we aim to determine the differential contributions of PEP and LVET in autonomic space by comparing measures of CAB and CAR that are calculated using each systolic time interval independently (i.e., comparing CAB_PEP to CAR_PEP and comparing CAB_LVET to CAR_LVET). If LVET and HRV measures represent chronotropic cardiac influence, and PEP represents inotropic cardiac influence, then CAB and CAR calculated using LVET should more accurately depict autonomic “space” compared to CAB and CAR calculated using PEP. Therefore, the following investigation examines the impact different systolic time intervals (PEP and LVET) can have on the association between CAB and CAR. We hypothesize that CAB and CAR calculated using PEP will be more closely associated compared to CAB and CAR calculated using LVET. Support for these hypotheses would suggest that PEP provides less of a distinction (or less autonomic space) between CAB and CAR compared to LVET. Thus, the current investigation evaluates the impact of chronotropic (LVET) verses inotropic (PEP) measures in both the calculation and validity of impedance derived measures of CAB and CAR.

## Methods

### Participants and Procedures

Participants were recruited via two methods: (a) an introductory level psychology course research pool, where students earned class credit for participating; and (b) outside of the research pool, with these individuals being compensated with cash. Data were pooled over three studies (*N* = 158, 107 females, 57 minorities, *M* age = 19.09, SD = 1.84, age range: 18–30). All participants were apparently healthy and did not readily present any mental or physical disorders.

We asked all participants not to smoke, undergo vigorous physical activity, or drink caffeine or alcohol in the six hours prior to the experiment. The methods of each study were approved by the institutional review board at The Ohio State University (IRB Protocol Number: 2012B0580) and followed the STROBE (Strengthening the Reporting of Observational studies in Epidemiology) checklist (Knottnerus and Tugwell, 2008),All participants gave written informed consent. All experimental sessions were conducted between 9am and 5pm. Prior to each session, participants were asked if they wanted to use the restroom and were given the opportunity to do so if necessary. In all studies, participants were placed in a soundproof experimental room that was held at room temperature (70 to 73 degrees Fahrenheit, or 21 to 23 degrees Celsius) and equipped with a camera and microphone for safety and instructional purposes as well as a high-definition TV (for stimuli presentation which did not occur in the present study). Participants were given a detailed explanation of the procedures that would take place without indicating the specific hypothesis under study or the manipulations applied. Electrocardiogram (ECG) leads were attached to the subjects and while in a separate control room, the experimenter led the subjects to the initial phases of the experiment. All participants first completed a 5-min baseline resting period, where participants, while spontaneously breathing, sat and viewed a blank, gray screen, and were instructed not to move or fall asleep while their cardiac activity was recorded via ECG. The “blank gray screen” contained no additional stimuli; the TV was turned on with a blank and “gray” screen so that participants were not able to view themselves via the reflection when powered off. The data for the present study was derived from this baseline period.

### Cardiovascular Measures

Cardiac data was recorded continuously throughout each experiment via a three-lead ECG with three additional leads for the ICG signal at a 1000 Hz sampling rate using a Mindware^TM^ 2000D (MW2000D) Impedance Cardiograph package. Electrodes were placed on the clavicle (1), ribs (2), lower back (1), lower sternum (1), notch of the throat (1), and back of the neck (1). Successive R-spikes were obtained from ECG recordings to calculate baseline HR and variability in these R-spikes was employed to calculate baseline HRV. Participants’ successive IBIs (in milliseconds) were extracted using Mindware^TM^ HRV Analysis software. IBIs were written in a text file and analyzed using Kubios HRV analysis package 2.0 (Tarvainen et al., 2014), allowing for the calculation of time- and frequency-domain indices of resting HRV (Task Force of the European Society of Cardiology, 1996). Artifacts within the R-to-R series were visually detected, and we applied an artifact correction level that would differentiate and remove artifacts (differing abnormal IBIs from the mean IBI). The detrending of time- and frequency-domain HRV measures was accomplished via the smoothness priors approach (see Tarvainen et al., 2014, for review). The root mean square of successive differences (RMSSD), measured in milliseconds, was calculated and is considered to be a stable (Li et al., 2009) and valid (Thayer et al., 2010a), time-domain measure of HRV. Autoregressive estimates were also calculated, yielding high-frequency power HRV (HF, 0.15–0.4 Hz; Thayer et al., 2010a). High-frequency peak values (HF hz) were obtained from a spectral-domain analysis as a measure of respiration frequency to control for potential bias (Thayer et al., 2002). Using Mindware^TM^ Impedance Cardiography Analysis software, mean PEPs and LVETs were also calculated (in milliseconds) in accordance with previously published guidelines (Sherwood et al., 1990). Specifically, Mindware Impedance Cardiography applies an algorithm that accurately identifies the Q peak (R onset) and the B point (start of the dz/dt peak) in the dz/dt wave form allowing for the calculation of both PEP and LVET for each individual (for more details, please see Berntson et al., 2004 and Lozano et al., 2007). As previously stated, CAB can be defined as a relative balance between parasympathetic and sympathetic nervous system activity. Therefore, it is calculated by subtracting the HRV measure for parasympathetic activity from the impedance measure for sympathetic activity, resulting in the relative difference in control between the two branches (Berntson et al., 2008). Conversely, CAR is defined as the total activity of both branches of the autonomic nervous system. Therefore, it is calculated by adding the HRV measure for sympathetic activity to the HRV measure for parasympathetic activity, resulting in a measure of total autonomic control (Berntson et al., 2008). Berntson et al. (2008) original formulas expressed the dynamics between the parasympathetic and sympathetic as *CAB* = *HFz – (−PEPz)* and *CAR* = *HFz* + *(−PEPz)*, which employ a chronotropic frequency-domain measure of parasympathetic activity (HF) and an inotropic impedance-derived measure of sympathetic activity (PEP). However, other research has identified RMSSD as an equally reliable time-domain measure of parasympathetic activity compared to HF (Penttilä et al., 2001; Balocchi et al., 2006; Sollers et al., 2007; Hill et al., 2009; Williams et al., 2017), while LVET has long been established as an impedance-derived index of sympathetic activity (Stemmler, 1993; Thayer and Uijtdehaage, 2001). Based on this information, the current conceptualizations of both CAB and CAR use either HF-HRV or RMSSD to reflect parasympathetic activity, and either PEP or LVET to reflect sympathetic activity. CAB and CAR were first calculated using the original parasympathetic measure from the Berntson et al. (2008) study, yielding four formulas for CAB and CAR that can be expressed as *CAB_H_PEP* = *HFz – (−PEPz)*, *CAR_H_PEP* = *(HFz)* + *(−PEPz)*, *CAB_H_LVET* = *HFz – (−LVETz)*, and *CAR_H_LVET* = *(HFz)* + *(−LVETz)*. CAB and CAR were then calculated using a time-domain measure of HRV to reflect parasympathetic activity, yielding four additional formulas that can be expressed as *CAB_R_PEP* = *RMSSDz – (−PEPz)*, *CAR_R_PEP* = *(RMSSDz)* + *(−PEPz)*, *CAB_R_LVET* = *RMSSDz – (−LVETz)*, and *CAR_R_LVET* = *(RMSSDz)* + *(−LVETz)*. In all calculations, z-scores are computed for the parasympathetic and sympathetic measures to account for disparities in their means and units of measurement, while the sympathetic measure is multiplied by −1 to reflect the fact that smaller values are indicative of greater sympathetic activity.

### Statistical Analyses

All statistical tests were conducted using SPSS (ver. 27, IBM Chicago, IL, United States). Zero-order correlations were performed between variables of interest including *z*-scored variables used to calculate CAB and CAR, as well as log-transformed variables used to calculate CAB and CAR. Confidence intervals (95%) were obtained for all correlation coefficients and are reported in brackets. Fisher’s *z*-to-*r* transformation was used to test differences between correlation coefficients. Statistics reported include Pearson’s r correlation values, 95% confidence intervals (in square brackets), and *p*-values.

Hierarchical regression analyses were also conducted to see whether CAR predicted CAB differentially based on calculations of the measures. Step one included covariates that were sex, age, body mass index, and race. An individual’s ethnicity can determine relative levels of resting HRV (Choi et al., 2006; Hill et al., 2015) and thus, was included as a covariate in applicable analyses (ethnicity coded as 0 = European American, 1 = Other). It is well-known that resting HRV decreases with age (e.g., Choi et al., 2006; Voss et al., 2015), therefore age was also included as a covariate. Body mass index was also included as previous research has shown that higher body mass index is associated with decreased resting HRV (e.g., Koenig et al., 2014; Molfino et al., 2009). Step two included respiration rate (HF Hz; Thayer et al., 2002). CAR calculated from either PEP (Model 1) or LVET (Model 2) were variables in their respective third step. Statistics reported include, change in *R*^2^ (Δ*R^2^)*, unstandardized beta (*b*) coefficients, standard errors (SE), 95% confidence intervals (in square brackets), partial correlation coefficients, and *p* values.

## Results

### Descriptive Statistics

Extreme outliers (+2SD) were removed, leaving a total sample of 158 participants (107 females, 57 minorities, *M*age = 19.09, *SDage* = 1.84, *M*BMI = 22.96, *SDBMI* = 3.77). Averages of raw scores for PEP (*M* = 118.20, *SD* = 10.71), LVET (*M* = 241.92, *SD* = 36.58), log-transformed HF (*M* = 6.65, *SD* = 0.93), and log-transformed RMSSD (*M* = 3.73, *SD* = 0.45), were obtained. We also reported averages for variables calculated using HF, which included CAB_H_PEP (*M* = 0.02, *SD* = 1.04), CAR_H_PEP (*M* = −0.13, *SD* = 1.02), CAB_H_LVET (*M* = 0.08, *SD* = 1.20), and CAR_H_LVET (*M* = −0.19, *SD* = 1.19), as well as variables calculated using RMSSD, which included CAB_R_PEP (*M* = −0.06, *SD* = 0.92), CAR_R_PEP (*M* = −0.20, *SD* = 0.86), CAB_R_LVET (*M* = 0.00, *SD* = 1.07), and CAR_R_LVET (*M* = −0.26, *SD* = 1.08). Please see Table 1 for descriptive statistics.

**TABLE 1:** Descriptive statistics of variables of interest.

	***M***	***SD***	***Range (min, max)***
Age	19.09	1.84	18.00, 30.00
BMI	22.96	3.77	14.98, 35.29
Respiration Rate	0.25	0.06	0.15, 0.38
RMSSD	45.90	19.12	9.90, 102.47
HF	36.61	19.08	2.13, 87.74
PEP	118.20	10.71	90.00, 140.00
LVET	241.92	36.58	128.00, 326.00
*ln*RMSSD	3.73	0.45	2.29, 4.63
*ln*HF	6.65	0.93	3.96, 8.87
*ln*PEP	4.77	0.09	4.50, 4.94
*ln*LVET	5.48	0.16	4.85, 5.79
*z*RMSSD	–0.13	0.79	−1.63, 2.22
*z*HF	–0.05	0.95	−1.77, 2.49
*-z*PEP	–0.07	0.40	−0.89, 0.99
*-z*LVET	–0.13	0.73	−1.81, 2.14
CAB_HF_PEP	0.02	1.04	−2.44, 2.78
CAR_HF_PEP	–0.13	1.02	−2.46, 2.30
CAB_HF_LVET	0.08	1.20	−2.25, 3.17
CAR_HF_LVET	–0.19	1.19	−2.62, 2.85
CAB_RMSSD_PEP	–0.06	0.92	−2.25, 2.51
CAR_RMSSD_PEP	–0.20	0.86	−2.14, 2.04
CAB_RMSSD_LVET	0.00	1.07	−2.83, 2.90
CAR_RMSSD_LVET	–0.26	1.08	−2.10, 2.80

*The table above includes means (M), standard deviations (SD), and the range (minimum, maximum) for raw scores of root mean square of successive differences (RMSSD), high frequency heart rate variability (HF), pre-ejection period (PEP), left ventricular ejection time (LVET), and log-transformed scores of RMSSD, HF, PEP, LVET, *z*-scored RMSSD, HF, PEP, LVET. It also includes M, SD and ranges for cardiac autonomic balance (CAB) calculated using HF, RMSSD, PEP, and LVET as well as cardiac autonomic regulation (CAR) using those same variables. *ln* = natural log-transformed; *z* = *z*-scored variable; −*z* = inverse of *z*-scored variable.*

### Zero-Order Correlations

**FIGURE 2:**
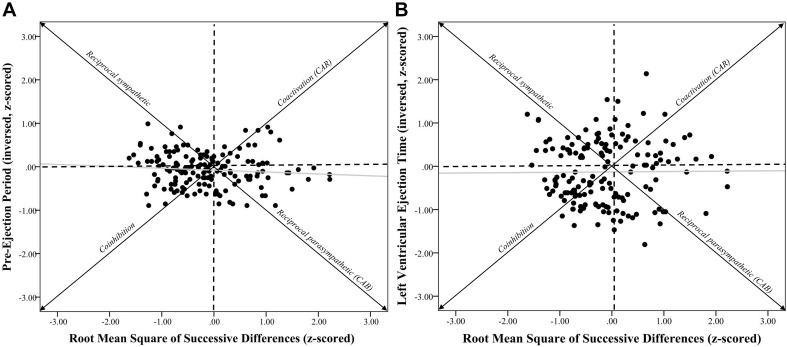
Systolic time intervals and root mean square of successive differences scatterplots. *Note*. (**A**) shows a scatterplot between pre-ejection periods (PEP *z*-scored and inversed, see section “METHODS” for details) and root mean square of successive differences (RMSSD-HRV, *z*-scored) (*r* = –0.09, *p* = 0.28). (**B**) shows a scatterplot between left ventricular ejection time (LVET inversed and z-scored) and RMSSD-HRV (*r* = 0.01, *p* = 0.91). Individuals in the coinhibition quadrant would show lower CAR scores, while individuals in the coactivation quadrant would show higher CAR scores. Individuals in the reciprocal sympathetic quadrant would show lower CAB scores, while individuals in the reciprocal parasympathetic quadrant would show higher CAB scores.

**FIGURE 3:**
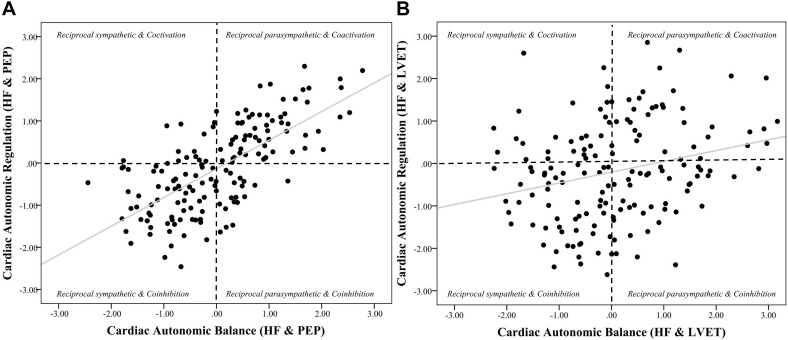
Scatterplots of cardiac autonomic balance and regulation computed using HF-HRV and both PEP and LVET. *Note.* (**A**) depicts the strong significant association between cardiac autonomic balance (CAB) and regulation (CAR) calculated using high frequency heart rate variability (HF) and pre-ejection periods (PEP) (*r* = 0.69, *p* < 0.001). (**B**) depicts a significantly weaker association between CAB and CAR calculated using left ventricular ejection time (LVET) (*r* = 0.26, *p* < 0.001). The correlation coefficient between CAR and CAB computed using PEP was significantly stronger than when computed using LVET (*z* = 5.12, *p* < 0.001).

**FIGURE 4:**
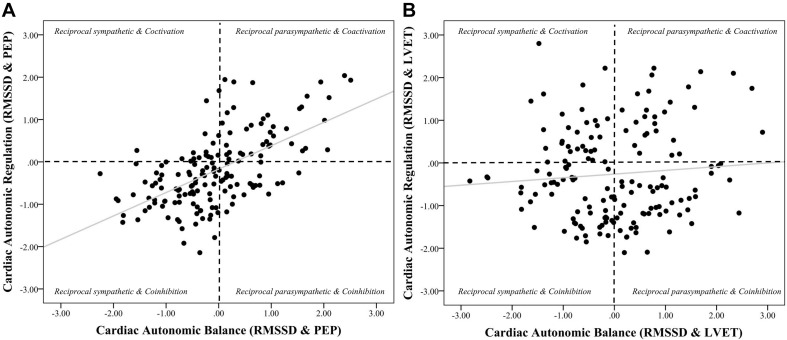
Scatterplots of cardiac autonomic balance and regulation computed using RMSSD-HRV and both PEP and LVET. *Note.* (**A**) depicts the strong significant association between cardiac autonomic balance (CAB) and regulation (CAR) calculated using high frequency heart rate variability (HF) and pre-ejection periods (PEP) (*r* = 0.59, *p* < 0.001). (**B**) depicts the lack of an association between CAB and CAR calculated using left ventricular ejection time (LVET) (*r* = 0.08, *p* = 0.28). The correlation coefficient between CAR and CAB computed using PEP was significantly stronger than when computed using LVET (*z* = 5.26, *p* < 0.001).

Zero-order correlational analyses were conducted (see Tables 2, 3) and plotted (see Figures 1–4) for various measures of HRV, impedance, CAB, and CAR.

**TABLE 2:** Zero-order correlations among variables used to calculate cardiac autonomic balance and cardiac autonomic regulation.

		**1**	**2**	**3**	**4**
1.	RMSSD*z*	–			
2.	HF*z*	0.44**	–		
3.	−PEP*z*	−0.09	−0.03	–	
4.	−LVET*z*	0.01	−0.01	0.36**	–

*Zero-order correlations between root mean square of successive differences (RMSSD), high frequency heart rate variability (HF), pre-ejection period (PEP), and left ventricular ejection time (LVET). These variables were used to calculate cardiac autonomic balance and cardiac autonomic regulation variables. Significant correlations are bolded; *z* = *z*-scored variable, −*z* = inverse of *z*-scored variable, ^∗∗^*p* < 0.01.*

**TABLE 3:** Zero-order correlations among CAB and CAR variables.

**A**	**1**	**2**	**3**	**4**
1. CAB_H_PEP	–			
2. CAR_H_PEP	0.69**	–		
3. CAB_H_LVET	0.82**	0.64**	–	
4. CAR_H_LVET.	64**0.81**	0.26**	–	
**B**	**1**	**2**	**3**	**4**
1. CAB_R_PEP	–			
2. CAR_R_PEP	0.59**	–		
3. CAB_R_LVET	0.77**	0.53**	–	
4. CAR_R_LVET	0.56**	0.77**	0.09	–

Table 3A shows zero-order correlations between cardiac autonomic balance (CAB) and cardiac autonomic regulation (CAR) variables that were calculated using high frequency heart rate variability (denoted as “*H*”) and pre-ejection period (PEP) or left ventricular ejection time (LVET), respectively. Table 3B shows CAB and CAR calculated using the root mean square of successive differences (denoted as “*R*”) and pre-ejection period (PEP) or left ventricular ejection time (LVET), respectively. Statistically significant correlations are bolded, **p < 0.01.

Zero-order correlational analyses were conducted (see Tables 2, 3). Results showed that there was a moderate, significant correlation between HF*z* and RMSSD*z* (*r* = 0.44, CI [0.31, 0.56], *p* < 0.001). Importantly, there was a significant strong correlation between *ln*HF and *ln*RMSSD (*r* = 0.90, CI [0.87, 0.93], *p* < 0.001). Results also showed a significant positive association between −PEP*z* and −LVET*z* (*r* = 0.36, CI [0.22, 0.49], *p* < 0.001).

Results showed that correlations between HF*z* and −PEP*z* (*r* = −0.03, CI [−0.19, 0.13], *p* = 0.73) as well as −LVET*z* (*r* = −0.01, CI [−0.17, 0.15], *p* = 0.90) were not statistically significant. Additionally, correlations between RMSSD*z* and −PEP*z* (*r* = −0.09, CI [−0.24, 0.07], *p* = 0.28) as well as −LVET*z* (*r* = 0.01, CI [−0.15, 0.17], *p* = 0.91) were also not significant.

Results revealed that there was a significant relationship between CAR_PEP and CAB_PEP calculated using both HF (*r* = 0.69, CI [0.60, 0.76], *p* < 0.001) and RMSSD (*r* = 0.59, CI [0.48, 0.68], *p* < 0.001). There was a significant correlation between CAB_LVET and CAR_LVET calculated using HF (*r* = 0.26, CI [0.11, 0.40], *p* < 0.001) but not RMSSD (*r* = 0.08, CI [−0.08, 0.23], *p* = 0.28). The correlation coefficient between CAB_PEP and CAR_PEP was significantly stronger compared to the correlation found between CAB_LVET and CAR_LVET for both HF (*z* = 5.12, *p* < 0.001) and RMSSD (*z* = 5.26, *p* < 0.001).

### Regression Analyses

#### Cardiac Autonomic Balance and Regulation Calculated Using HF

Regression analyses (see Table 4) revealed that CAR_PEP significantly predicted 49.9% of the variance in CAB_PEP (Δ*R*^2^ = 0.40, *b* = 0.69, *SE* = 0.06, *rpartial* = 0.67, CI [0.56, 0.81], *p* < 0.001). In contrast, CAR_LVET significantly predicted 13.0% of the variance in CAB_LVET (Δ*R*^2^ = 0.04, *b* = 0.22, *SE* = 0.08, *rpartial* = 0.22, CI [0.06, 0.38], *p* = 0.01). The association between CAR_PEP and CAB_PEP (*rpartial* = 0.67) was significantly stronger (*z* = 5.17, *p* < 0.001) compared to the association between CAR_LVET and CAB_LVET (*rpartial* = 0.22).

**TABLE 4:** Hierarchical regression analyses calculated from HF, PEP, LVET.

	**Cardiac Autonomic Balance (PEP)**						**Cardiac Autonomic Balance (LVET)**					
**Predictor Step**	**Δ*R*^2^**	***b***	***SE***	***p***	**95%CI**	***rpartial***	**Δ*R*^2^**	***b***	***SE***	***p***	**95%CI**	***rpartial***
*R*^2^		0.50**						0.13**				
Sex		–0.02	0.13	0.89	[−0.28, 0.25]	–0.01		0.21	0.20	0.30	[−0.19, 0.61]	0.09
Age		0.00	0.03	0.93	[−0.06, 0.07]	0.01		0.01	0.05	0.91	[−0.09, 0.11]	0.01
BMI		–0.02	0.02	0.35	[−0.05, 0.02]	–0.08		–0.01	0.03	0.57	[−0.07, 0.04]	–0.05
Race		0.06	0.13	0.62	[−0.19, 0.32]	0.04		0.09	0.20	0.63	[−0.29, 0.48]	0.04
Respiration												
Rate		1.90	0.98	0.06	[−0.04, 3.85]	0.16		4.23**	1.48	0.01	[1.30, 7.16]	0.23
*CAR_HF_PEP*	0.40**	0.69**	0.06	< 0.001	[0.56, 0.81]	0.67		–	–	–	–	–
*CAR_HF_LVET*		–	–	–	–	–	0.04**	0.22**	0.08	0.01	[0.06, 0.38]	0.22

The table above shows the unstandardized beta coefficients (b) with associated significant levels at each step in the regression model. Regression analyses (left) of cardiac autonomic regulation (CAR) calculated from pre-ejection period (PEP) and high frequency heart rate variability (HF) predicting cardiac autonomic balance (CAB) calculated from PEP and HF. Regression analyses (right) of CAR calculated from left ventricular ejection time (LVET) and HF predicting CAB calculated from LVET and HF, **p < 0.01.

#### Cardiac Autonomic Balance and Regulation Calculated Using RMSSD

For CAR and CAB computed using RMSSD (see Table 5), results showed that CAR_PEP significantly predicted 37.4% of the variance in CAB_PEP (Δ*R*^2^ = 0.34, *b* = 0.63, *SE* = 0.07, *rpartial* = 0.59, CI [0.49, 0.77], *p* < 0.001). In contrast, CAR_LVET did not significantly predict CAB_LVET (Δ*R*^2^ = 0.01, *b* = 0.09, *SE* = 0.08, *rpartial* = 0.10, CI [−0.06, 0.25], *p* = 0.24) and only explained 5.5% of the variance in CAB_LVET. The association between CAR_PEP and CAB_PEP (*rpartial* = 0.59) was significantly stronger (*z* = 5.08, *p* < 0.001) compared to the association between CAR_LVET and CAB_LVET (*rpartial* = 0.10).

**TABLE 5:** Hierarchical regression analyses calculated from RMSSD, PEP, LVET.

	**Cardiac Autonomic Balance (PEP)**						**Cardiac Autonomic Balance (LVET)**					
**Predictor Step**	**Δ*R*^2^**	***b***	***SE***	***p***	**95%CI**	***rpartial***	**Δ*R*^2^**	***b***	***SE***	***p***	**95%CI**	***rpartial***
*R*^2^		0.37**						0.06				
Sex		–0.16	0.13	0.22	[−0.42, 0.10]	–0.10		–0.15	0.19	0.43	[−0.52, 0.22]	–0.06
Age		–0.01	0.03	0.75	[−0.08, 0.06]	–0.03		–0.03	0.05	0.57	[−0.12, 0.07]	–0.05
BMI		–0.03	0.02	0.10	[−0.06, 0.01]	–0.14		–0.04	0.02	0.08	[−0.09, 0.00]	–0.14
Race		0.01	0.13	0.91	[−0.23, 0.26]	0.01		–0.02	0.18	0.91	[−0.38, 0.34]	–0.01
Respiration												
Rate		1.31	0.96	0.17	[−0.59, 3.21]	0.11		2.63	1.37	0.06	[−0.08, 5.34]	0.15
*CAR_R_PEP*	0.34**	0.63**	0.07	< 0.001	[0.49, 0.77]	0.59		–	–	–	–	–
*CAR_R_LVET*		–	–	–	–	–	0.01	0.09	0.08	0.24	[−0.06, 0.25]	0.10

The table above shows the unstandardized beta coefficients (b) with associated significant levels at each step in the regression model. Regression analyses (left) of cardiac autonomic regulation (CAR) calculated from pre-ejection period (PEP) and root mean square of successive differences (RMSSD; denoted as “*R*”) predicting cardiac autonomic balance (CAB) calculated from PEP and RMSSD. Regression analyses (right) of CAR calculated from left ventricular ejection time (LVET) and RMSSD predicting CAB calculated from LVET and RMSSD, **p < 0.01.

## Discussion

The purpose of the current investigation was to evaluate PEP and LVET in the calculation of CAB and CAR in order to determine which systolic time interval provided CAB and CAR with optimal autonomic space. Our results showed the association between z-transformed HRV (HF and RMSSD) and both PEP and LVET to be near zero, however, HRV and LVET appear to show better space given the spread of data points. Importantly, there was a stronger association between CAB and CAR when calculated using PEP compared to LVET, which show little (calculated using HF) to no (calculated using RMSSD) association between CAB and CAR. In other words, when calculated using PEP, individuals higher in CAB are more likely to be higher in CAR. In contrast when calculated using LVET, the association between CAB and CAR is significantly lower, and when calculated using RMSSD, is negligible. Taken together, these data suggest that LVET provides better autonomic space compared to PEP when paired with HRV in the calculation of CAB and CAR. Furthermore, we highlight that the association between CAB and CAR computed using RMSSD and LVET was not significant. This may further suggest RMSSD as a better measure for the calculation of CAB and CAR.

Cardiac autonomic balance (CAB) and CAR are designed to capture opposing modes of autonomic activity, with CAB reflecting a propensity toward the dominance of either the sympathetic or parasympathetic branch, and CAR reflecting the co-activation or co-inhibition of both branches. With this in mind, our findings may suggest that CAR and CAB calculated using PEP may not sufficiently reflect these functional differences, as indicated by their strong agreement. Given that the associations seen between PEP-derived CAR and CAB remain strong regardless of which chronotropic HRV measure is used in their calculations, it is likely these associations are the result of PEP failing to provide adequate coverage of autonomic space. One potential reason behind this is that, as previously mentioned, PEP represents an inotropic measure of sympathetic activity, influencing myocardial contractility at the atrioventricular (AV) node of the heart. In contrast, LVET shares a functional foundation with chronotropic measures HF and RMSSD (Stemmler, 1993). When CAB and CAR are calculated using LVET we see that the two measures are in little-to-no association; especially when they are calculated using RMSSD. This suggests that while there may be circumstances under which CAB and CAR may be significantly associated when calculated using LVET (i.e., using HF), it is significantly weaker compared to using PEP, and not significant when calculated using RMSSD. Overall, these results suggest that LVET-derived CAB and CAR represent more distinct patterns of autonomic activity due to LVET providing better autonomic space compared to PEP. Furthermore, these results also suggest that chronotropic time-domain measures of HRV (i.e., RMSSD) and impedance cardiography may be superior indices of parasympathetic and sympathetic activity when calculating CAB and CAR. A potential reason for this pattern may be due to time-domain HRV measures (especially RMSSD) being more resistant to violations of stationarity compared to frequency domain measures (Tarvainen et al., 2002).

### Implications

The psychophysiological connection between the autonomic nervous and cardiovascular systems continues to be at the forefront of health research. An imbalance or dysregulation of this relationship is of particular interest, given its association with stress (Wulsin et al., 2018), psychopathologies (Thayer and Brosschot, 2005), difficulties in emotion regulation (Williams et al., 2015), cardiovascular disease risk factors (Thayer et al., 2010b), and all-cause mortality (Thayer and Sternberg, 2006). As such, special attention should be given to the methods and formulas designed to quantify this relationship, especially in regards to the balance and regulation of the parasympathetic and sympathetic branches of the autonomic nervous system. The development of CAB and CAR has proven to be a vital step toward the conceptualization of cardiac autonomic activity, with both serving as valid and reliable indices of the dynamic between the parasympathetic and sympathetic nervous systems in several studies examining mental (Gump et al., 2011; Kreibig et al., 2012; Bylsma et al., 2015) and physical (Berntson et al., 2008; Singh et al., 2009; Vrijkotte et al., 2015) health. However, our data suggests that the calculation of these measures can be adjusted to build upon their efficacy as markers of psychophysiological health.

Additionally, with research showing that the health-related significance of various states of cardiac autonomic control of the heart can vary across different psychological stressors and pharmacological blockades (Carlsson et al., 1977; Stemmler, 1993; Berntson et al., 1994a), calculating CAB and CAR using cardiac autonomic measures with a shared functional foundation may be especially important in accurately classifying individuals and their respective cardiovascular states.

From a methodological perspective, calculating CAB and CAR using LVET may be beneficial for increasing their precision in predicting cardiovascular functioning. As previously mentioned, both parasympathetic and sympathetic influences can have differential effects on the heart depending on the effector tissue involved; even when both systems are active. For example, autonomic influences involved in the control of heart rate at the SA node (i.e., chronotropy) tend to be dependent on the level of background sympathetic activity, with higher levels of sympathetic activation resulting in greater decreases in heart rate associated with a given parasympathetic stimulus (a phenomenon known as accentuated antagonism; Levy and Zieske, 1969). Similarly, autonomic influences involved in cardiac contractility at the AV node (i.e., inotropy) are also dependent on the level of background sympathetic activity. While parasympathetic influence over contractility is negligible with low or no sympathetic activation, increases in sympathetic activity result in marked, non-algebraically additive decreases in contractility (Levy, 1997). Whereas studies tend to calculate CAB and CAR using different chronotropic measures such as HF (Singh et al., 2009), RMSSD (Williams et al., 2017), and respiratory sinus arrythmia (Kreibig et al., 2012), these and other studies almost exclusively use the inotropic measure of PEP to index sympathetic activity as opposed to more appropriate chronotropic sympathetic measures like LVET. Our results suggest that CAB and CAR show dependency when calculated using PEP irrespective of the HRV index used. Of course, this should not be the case, given that these measures reflect different states of cardiovascular functioning, albeit through similar modes of autonomic activity (e.g., coactivation of the parasympathetic and sympathetic branches). Therefore, it is possible that the results of studies that have used PEP-derived measures of CAB and CAR to capture autonomic activity may be limited in their accuracy or interpretation as they relate to cardiac autonomic activity, and our results suggest that using LVET in place of PEP may yield more appropriate results. Indeed, the association between CAB variables and CAR variables derived using different HRV measures and systolic time intervals show considerably high correlations (*r*’s between.6 and.8), however these correlations are far from perfect as one might expect, and thus can have a considerable impact on both data and results.

### Limitations and Future Directions

The current study is not without its limitations. The first limitation is that the sample largely consists of college students, and therefore the results may not be generalizable to all age groups. While we are confident that the present relationships seen between the various calculations of CAB and CAR would be present across all ages, future research should collect HRV and impedance data and conduct similar analyses to confirm this. To this end, when using consistent chronotropic measures to compute CAB and CAR in our young and healthy sample of individuals, true variation within autonomic space is revealed. In contrast, the Berntson et al. (2008) report showed similar variation using PEP and HF-HRV, however their sample of individuals were significantly older (ranging between 50 and 68 years) and some showed cardiovascular diseases (e.g., diabetes). Thus, it would be important to understand the differential impact of calculating CAB and CAR using LVET/PEP in both older and younger individuals, in addition in those who may show cardiovascular complications.

Additionally, although we did ask participants to smoke in the hours prior to the study, we did not ask if they were regular smokers, which may or may not have had an influence on general cardiovascular and respiratory functioning in select individuals. Another limitation is that we did not measure respiration via direct methods (e.g., using a transducer belt or counting thoracoabdominal movements) to ensure that participants had a breathing rate of at least nine respirations per minute, which could have influenced our current results. However, RMSSD has been shown to be resistant to respiratory influence following detrending and thus, results surrounding RMSSD are relatively free of respiratory influence (Lewis et al., 2012; Laborde et al., 2017). Lastly, our results may be limited by a lack of a pharmacological blockade to more accurately verify patterns of autonomic activity. However, as previously stated, sympathetic and parasympathetic influences on the heart differ based on the effector tissue involved, even when both branches are active. Thus, introducing a blockade to either one of these influences may effectively eliminate an important piece of the physiological puzzle. Additionally, we are not proposing a new method of indexing autonomic activity, but rather offering a more precise method of calculating CAB and CAR, which has already been verified via blockade studies (Berntson et al., 1994a; Cacioppo et al., 1994).

Future research aiming to replicate our research should also attempt to record HRV and impedance data over different or extended time periods, such as comparing CAB and CAR measures during the day and at night. Lastly, it may be beneficial for future studies to examine sex differences in the various calculations of CAB and CAR, as our current sample is predominantly female, and a recent meta-analysis on sex differences in HRV determined that women have higher vagal tone compared to males (Koenig and Thayer, 2016). Moreover, a recent investigation found that the association between HRV and heart rate was not equal between women and men, suggesting a differential influence of autonomic activity on heart chronotropy based on sex (Williams et al., in prep).

## Conclusion

As researchers continue to explore the physiological connection between the autonomic nervous system and cardiovascular functioning, special consideration should be given to how the dynamic between the parasympathetic and sympathetic branches are indexed and interpreted. Our data show that measures of CAR calculated from PEP significantly correlate with and predict measures of CAB calculated using PEP. Conversely, computed using LVET, CAR shows a significantly weaker association using HF-HRV and no association using RMSSD-HRV. The current study provides evidence suggesting that the chronotropic systolic time interval LVET provides better autonomic space compared to the inotropic measure PEP, making it the superior index of sympathetic activity in the calculation of CAB and CAR.

## Data Availability Statement

The raw data supporting the conclusions of this article will be made available by the authors, without undue reservation.

## Ethics Statement

The studies involving human participants were reviewed and approved by The Ohio State University Institutional Review Board. The patients/participants provided their written informed consent to participate in this study.

## Author Contributions

All authors listed have made a substantial, direct and intellectual contribution to the work, and approved it for publication. Author order was agreed upon prior to the writing of the manuscript, with CW serving as first author and DW serving as senior author.

## Conflict of Interest

The authors declare that the research was conducted in the absence of any commercial or financial relationships that could be construed as a potential conflict of interest.
